# Towards Safety Improvement of Measurement and Control Signals Transmission in Marine Environment †

**DOI:** 10.3390/s20061668

**Published:** 2020-03-17

**Authors:** Romuald Masnicki, Janusz Mindykowski, Przemyslaw Grala

**Affiliations:** 1 Faculty of Electrical Engineering, Gdynia Maritime University, Morska 81-87, 81–225 Gdynia, Poland; j.mindykowski@we.umg.edu.pl; 2 Faculty of Electrical Engineering, Candidate for a degree from Gdynia Maritime University, Morska 81-87, 81–225 Gdynia, Poland; przgrala@gmail.com

**Keywords:** measurement and control, marine environment, wireless sensor network, signal transmission, safety improvement, ZigBee, WiFi

## Abstract

This paper is dedicated to some aspects of maritime on-board safety improvement. The contribution of this article is a proposal to extend wireless communication on the ship, supported by examples of solutions that have not previously been used on board. Analyzed aspects concern the measurement and control signal transmission in hostile marine environment. A problem to solve is formulated, how to reduce a negative impact of this environment on effectiveness and reliability of maritime on board communication. The proposed ways for solving a problem under consideration cover recommendations concerning some aspects of ship construction and implementation of wireless sensor network. The main topic faced by the paper is concentrated on case study-based ZigBee as well as WiFi networks implementation in the area of the acquisition of data from sensors and measuring transducers connected to the terminal network. The exemplary implementation of ZigBee network, elaborated in Gdynia Maritime University is related, firstly, to the configuration of a simple wireless measurement and control channel, and secondly, to wireless communication channel supported by autonomously working microprocessor measurement and control system. WiFi networks were also tested in the same area of application. Finally, some concluding remarks are formulated.

## 1. Introduction

The core information of this paper is based on a recently published paper [1], describing some aspects of maritime on-board safety improvement mainly case study-based ZigBee network implementation in ship technology. Nowadays ships are very sophisticated technical objects operating in marine environment. A modern automated cargo ship as an object of measurement and control can be described by a complex structure of four main systems: navigation, power, cargo and crew living conditions [2]. Along with increasing pollution and the greenhouse effect on a global scale, new challenges arise in ship technology [3] - designing new systems reducing the environmental burden of ship operation. All these systems require supervision by measurement and control sensor networks for ensuring safe and effective ship operation [4,5,6,7], including its fundamental mission: transportation of cargo and people. In this context, a harsh marine environment is a special area considering working conditions of people and devices installed on a ship. Many factors contribute to this state of affairs, including [5]:

- high relative humidity in presence of sea salt,
- high temperature prevailing in many rooms of the ship,
- accumulation of devices, including electrical ones, in a small space,
- relatively frequent emergency situations and the related need to work in cases requiring immediate action,
- work in conditions of limited space,
- monotony and routine in the performance of work,
- rolling, pitching and tilting the ship,
- noise and vibrations.

Safety issues of crew and ship equipment play key role in the operation of the ship. Taking into account a negative impact of hostile marine environment on day-to-day ship operations, efficient ship internal communication is essential for maritime safety. Safety includes the reliable exchange of measurement and control information between people, between people and devices, as well as between the devices themselves. Analyzing the possibilities of safety improvement of aforementioned information exchange, two aspects should be taken into account: connectivity of related sensor networks and technology for data transmission. 

A large number of cable connections on a ship is a challenge for ship designers [8]. The scope of ship cables includes: power, external and internal communication, instrumentation, data transmission, measurement and control as well as monitoring and signaling. The cables are deployed in a complex and extensive network of cable trays. The cable connections creating communication channels must grant a whole range of requirements related to resistance of signals transmitting information against disturbances and correctness of transmission. In many applications, a special mechanical construction of cable is required for affirmation of immunity on harmful materials (as oil, gases, salt and moisture) or mechanical factors (as tension, vibrations, friction, etc.), particularly, when the connections are applied among movable devices. Interestingly, ship electrical systems break down more often from the failure of some mechanical (rather than electrical) connection issue, like a parted wire splice, a frayed wire, a loose connection, or a missing or loose screw. Each of them can disturb the communication data flow, causing equipment to not work at all or to operate at less than optimum levels. 

Some of the above-mentioned problems with cable connections, especially in the areas of measurement, control, monitoring and signaling, can be solved by using wireless technologies for data transmission. Similarly, the wireless communication seems to be reasonable application, where either the object is situated in an inaccessible place or where the structure of the cable connections is expensive. Since deploying full-scale wired sensor networks on a ship leads to complexity and high costs, Cho et al. proposed a Wireless Sensor Network (WSN) solution [9]. Fundamentals and many interesting information related to this solution namely WSN technology, can be found in [10]. 

On the other hand, the Internet related technologies, including wireless WiFi, are beginning to be introduced on board the ships, mainly as an information and entertainment medium for ship’s crew as well as passengers use. This applies mostly the cable Ethernet. The WiFi is used only in some public places on the ship, e.g. in the crew mess room. On cruise ships, there are specific zones that offer passengers Internet connections from anywhere in the world. Some ships also have marked WiFi zones that offer wireless internet usage for everyone on board.

An added value of this paper concerns the design and implementation of sample ZigBee and WiFi networks in modified configurations that can be used on a ship in measurement and control systems. The study was developed at Gdynia Maritime University (GMU). 

The ZigBee implementation consists of two cases: firstly, as an application dedicated to valve control, and secondly, as wireless communication channel supported by autonomously working microprocessor measurement and control system cooperating with the ZigBee network. At present, the distributed microprocessor systems are usually used for implementation of the measurement, control, monitoring and safety functions related to previously mentioned ship systems, so the presented solutions are representative for co-operation with the ZigBee network. 

Research on the physical model of the WiFi communication channel shows that this network standard also applies to the ship’s environment for measurement and control applications. 

In all conducted experiments, specific properties of both network standards were used, not yet included in the results described in the literature, used to enable signal transmission through steel bulkheads ubiquitous on ships.

The experiments carried out were based on the physical models of the authors of the ZigBee network (prototypes of the ZigBee terminal device and microprocessor measuring and control devices were developed) and the WiFi communication channel, supervised by applications in the NI LabVIEW (National Instruments, Laboratory Virtual Instrument Engineering Workbench) environment. 

Following the introduction, the rest of this article is structured as follows. Section 2 shortly characterizes a state of the art in the area of maritime on board communication. In Section 3, a problem statement and a proposal of its solution is shown. In Section 4, exemplary peripheral devices of wireless network elaborated in Gdynia Maritime University are presented and described. Section 5 shortly presents the carried out research and explain obtained results, as well as some comments on the integration of network data as part of wireless network research. Finally, concluding remarks and future works directions are formulated.

## 2. Maritime on Board Communication: State of Art

A conventional ship area network (SAN) has the functionality of a remote control and autonomous management of various sensors and instruments embedded or boarded on a ship. The main operations of on-board systems embedded in a variety of devices and instruments, detection and control of systems as well as data collection and management are key to safety. These operations take place in many parts of the ship, from the engine room, bridge, administrative staff, and even off the ship. The conventional SAN’s hierarchy, in accordance with the international standards, is usually organized as a single network with three functional layers [11]: Integrated Ship Control (ISC) layer, Process layer and Instrument layer. This network delivers main operations such as sensing and control shipboard systems and management of crucial information for safety and navigation. In last decade, SAN has integrated with navigation system on board using National Marine Electronics Association (NMEA) 2000 standard [11,12]. SAN is mainly based on wired networks, such as dedicated connections or instrument networks, among others analog standard 4–20 mA, cable Ethernet or Controller Area Network (CAN). WiMedia technology was proposed for wireless communication between devices operating in the ISC layer and the Instrument layer [12]. Wireless communication takes place only inside selected compartments on the ship.

Even if most ship owners nowadays have only a monitoring system covering very essential equipment based on wired sensor networks, and a personnel tracking system is seldom in place, the concept of hybrid network (both wired and wireless) or wireless network is more and more frequently applied in many industries, including ship applications. A wireless sensor network consists of spatially distributed autonomous devices using sensors to monitor physical or environmental conditions, such as temperature, pressure, vibration, etc. at different locations, as well as specific automation components in control operations. With the wireless communication technologies we can easily make so far inaccessible data visible, moreover, the next wireless applications, among others, in industrial automation and marine technology are expected. 

Transferring data over a wireless network requires three components: a radio signal, a fixed data format, and a network structure. Each of the elements works independently of the others, so to create a wireless network all three elements must be defined. Each wireless broadband network uses a different set of radio waves, data formats, and network structure. In the last years, several techniques of wireless exchange of information have been developed (e.g. Bluetooth, WiFi, WiMAX, ZigBee, GSM, UMTS, etc.). 

The main engine or other equipment, the ship rooms and compartments walls, as well as the passengers’ movements (metallic door closing and opening), can affect the quality of wireless communication. This environment has a specific metallic structure which makes the wireless communication more difficult than in other classical indoor and outdoor environments. 

Bu-Geun et al. authors have conducted ZigBee measurements on the passenger deck of a ship and a small WSN has been deployed between the main engine room and the control room [13]. Next, a WSN has also been tested successfully in the main engine room of a ship [14]. Finally, a WSN on board a ferry moored in the harbor has been tested [15]. All these measurements have been carried out when the ships were moored at port. To date of study [16], no experiments have been carried out during ship operation. To complete hitherto existing state of the art, Kdouh et al. authors studied the possibility of replacing a wired shipboard monitoring system by a WSN technology [16]. 

Two types of experiments based on dedicated measurement setup [16] have been carried out on board a ferry-type boat during sailings and stopovers. Measurement setup is discussed in the wake of characteristics of considered environments and the technology used for the measurement campaigns. Aforementioned characteristics describe the measurement sites properties and localization and used technology based on IEEE 802.15.4 standard as well as define related frequency band [17]. The equipment this standard is operating in the 2,4 GHz ISM (Industrial, Scientific and Medical) frequency band. 

The first experiment [16] consist of point-to-point measurements using ZigBee-based equipment and the second one consists of deploying and testing a WSN on board of ferry. Point-to-point communication tests covered communication between nodes placed in the same room, communication between nodes placed in adjacent rooms, and communication between nodes placed in adjacent decks. These tests have proved that communication becomes impossible when two watertight doors between the two communicating nodes are closed. Aforementioned experiments [16] are one of the first tests conducted on board a ferry during sailings and under realistic condition. The author has mentioned about using the hybrid sensor network to the ship application mainly for tracking the connectivity challenges and for increasing system reliability. Therefore, in addition to wires, a proposal to integrate the sensor network with other more established or to-be-established networks, such as a WiFi based mesh network, the crew/passenger network, a personnel tracking system and the global Internet was presented. Nevertheless, a later on, based on some different sources, the author of the paper [18] was stated, that although feasibility of WSN technology in ship applications can be justified from next examples from the literature (among others [16] and [19]), real deployments still meet difficulties. Although there are many networks functioning in industry, a lot of devices are still not connected to any network, because the cabling engineering is very costly. Additionally, wireless connection devices usually comprise the ADC converters on board, so data acquisition becomes more homogeneous. Digital data is transmitted to PC (as a base station), so the problem of conversion of analog information into its digital representation on the entrance to PC disappears. It is worth adding that in [18] it was stated: “as we know that neither a wired nor wireless solution of deploying sensor networks on board will not work well alone to combine their strength together becomes on intuitive thought”. This idea, which in fact refers to hybrid sensor networks, was implemented in [20] and [21]. On the basis of aforementioned short overview of the recent literature, the most relevant findings have been extracted and shortly commented on. In consequence, a list of main topics and key achievements appearing in the last years in papers dedicated to area of maritime on board communication is presented below (Table 1).

All cited studies confirmed that the communication on the ship, in individual rooms and compartments, can be conducted similarly to home conditions, also during ship operation at sea. The common feature of the cited research is that the authors did not tried to utilize the very beneficial properties of the ZigBee network: the ability to transfer data across multiple nodes (using multiple ZigBee Routers) to eliminate signal suppression on steel partitions on a ship. 

There is no data available regarding implementation of the WiFi network on the ship for measurement and control applications.

## 3. Problem Statement and Proposal of its Solution

**Figure 1:**
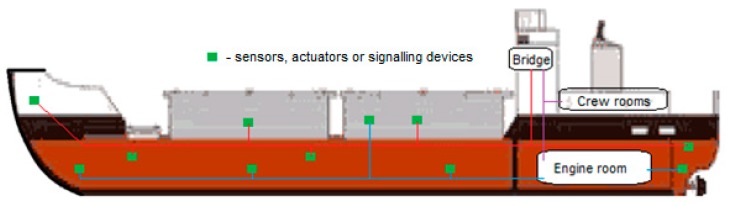
Overview sketch of communication connections on the ship.

Possibilities of safety improvement on board ships with regards to information exchange depend mainly on the communication effectiveness and reliability and are limited by two principal problems: connectivity of related sensor networks and technology for data transmission. Bearing in mind that communication of a ship can be categorized on the route between source and destination, usually understood as the cases: ship-to-shore, ship-to-ship and the on board case, in this paper the main focus is concentrated on the issue of maritime communication on board (Figure 1). This case provides basic data for the other two cases. A problem is formulated to solve how to reduce the influence of disturbing factors for effectiveness and reliability of maritime on board communication based on the implementation of a wireless sensor network solution.

**Figure 2:**
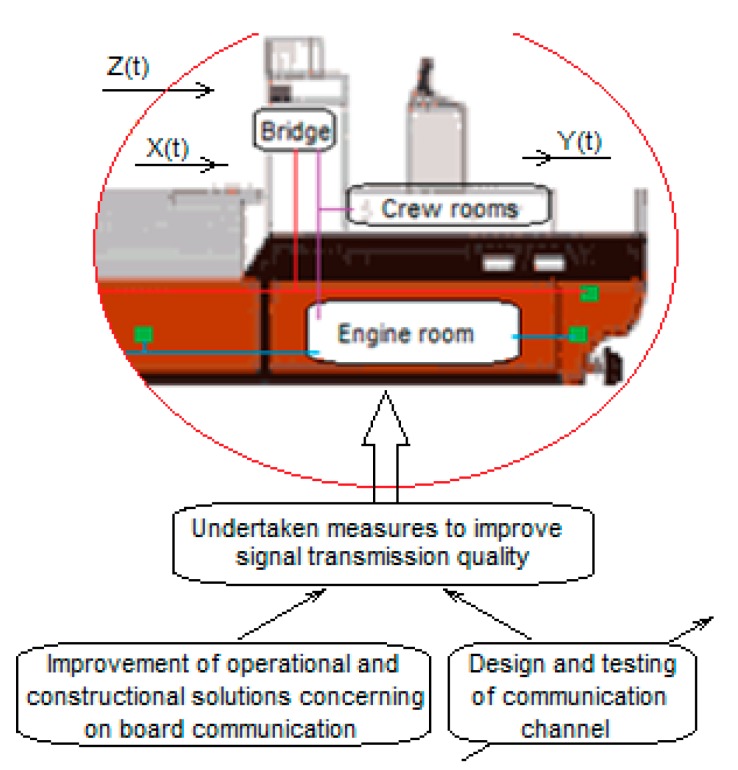
A block diagram of the performed investigation based on the case study-based ZigBee and WiFi networks implementation, where: X(t) – transmitted on board communication signals, Yo(t),– received on board communication signal, Z(t) – disturbances affecting the transmitted signals.

To improve the safety of crew and ship operations, two ways are proposed consisting of a robust configuration of a wireless measurement and control sensor network and the design and testing of a prototype of a reliable communication channel. A block diagram of the performed investigation, based on the case study–based network implementation, is shown in Figure 2. 

The undertaken measures for improving the quality of signal transmission in the case under investigation cover consideration of two aspects: partitions on board the ship and appropriate choice of a wireless network configuration. 

A significant reduction of the hostile influence of bulkheads and watertight doors on decreasing the power of received radio signals and their degradation can be obtained for installation of repeater nodes in dedicated tunnels in the partition walls. In case of the ZigBee network, the function of network self-organization as well as the ZigBee Routers (as integral devices of ZigBee network) are very useful in configuring the ship’s network. These features were used in the conducted research. The basic application of WiFi is multimedia access, via an Access Point (AP) device, to shared information on the Internet, such as website databases and e-mail, as well as communication within the home wireless LAN (Local Area Network). WiFi networks for these applications are also increasingly used on board ships. However, various WIFI repeaters have recently become available, with good quality and performance. Their use in ship conditions in Wi-Fi networks allows technicians to build distributed measurement and control systems.

### 3.1. Metal Compartments and Partitions on the Ship

**Figure 3:**
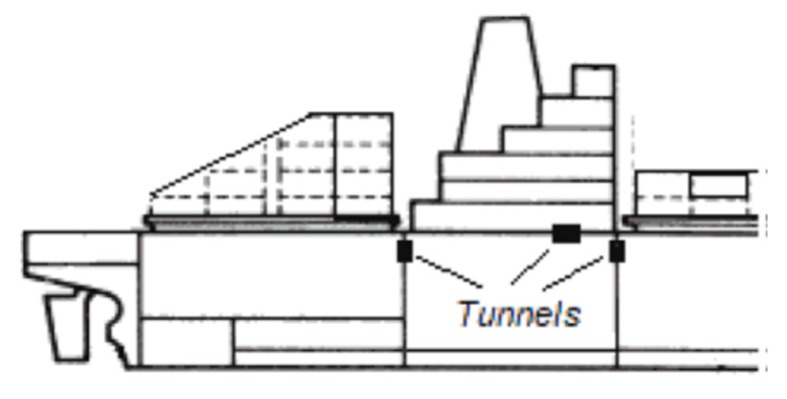
Wireless communication tunnels.

The wireless communication on board vessels is limited by several factors. Metallic structure of bulkheads and watertight doors severely decrease the power of received radio signals. Moreover, due to the metallic environments on board ships, propagation effects like the multipath can be a serious cause of received signal degradation [9]. The solution can be the installation of repeater nodes to overcome metal obstacles. They can be installed in dedicated tunnels in the partition walls (Figure 3). These tunnels help solve the problems of the aforementioned limitations in wireless communication by metal structures on the ship.

### 3.2. ZigBee Network Self-Organization of Communication

**Figure 4:**
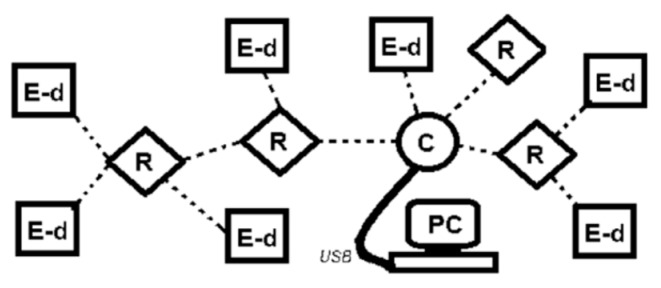
A ZigBee mesh network.

ZigBee wireless network. ZigBee based networks are characterized by low power consumption, low throughputs (up to 250 kbps) and range between nodes of 100 m. Typical applications are sensor networks, home automation, alarm systems, and monitoring systems. The ZigBee specification for the lower PHY and MAC layers uses the IEEE 802.15.4 standard, which provides wireless transmission in ISM frequency bands. The complete ZigBee mesh network equipment consists of three types of devices: Coordinator (C), Router (R) and End Device (E-d) (Figure 4). The Coordinator is mainly responsible for organizing and maintaining the network and also provides an interface in communication with the central processing unit (PC). Routers (also acting as repeaters) as intermediary network nodes, responsible for data routing, provide access to End Devices. The End Devices, connected for example with a measuring transducer, send measurement data to the Router (or directly to the Coordinator, if accessible). Among others, a ZigBee mesh network can automatically and seamlessly configure connections between End Devices and Coordinator, continually seek opportunities to improve their own performance and efficiency as well as automatically detect, diagnose, and repair localized software and hardware problems. In ship condition, a network self-organization property is particularly important, because of redundancy needs.

### 3.3. WiFi Network Implementation on The Ship

Wireless WiFi networks, along with wired Ethernet networks, are currently the most popular technologies used to build a LAN. The LAN, designed to facilitate easy and efficient exchange and access to data, allows computers and devices on the network to share software, files and hardware between connected nodes. WiFi is a standard developed by the IEEE, which defines a set of standards and specifications for wireless networks under the name "IEEE 802.11" [22]. Wifi networks, like ZigBee, work in ISM bands.

**Figure 5:**
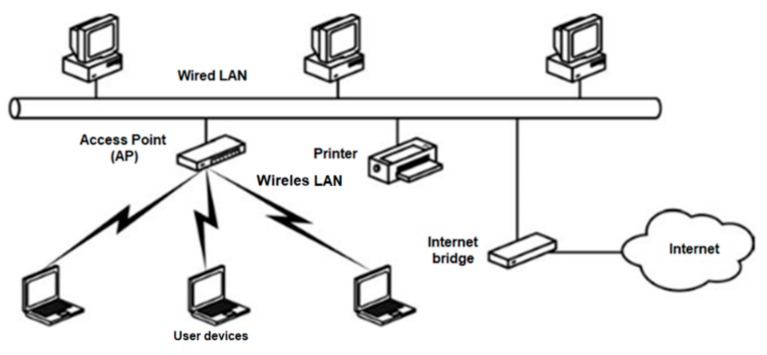
LAN network.

The basic LAN configuration is shown in Figure 5. Typically, two type devices are used in WiFi network: an access point (AP) and user devices. The wireless part of the LAN cannot operate in the ship’s state in the configuration presented, therefore its modification is necessary. Unlike the ZigBee, in typical configuration of WiFi network there is no mechanism for transmitting signals through metal partitions on a ship and the WiFi network is not self-organizing. However, it provides much wider bandwidth and higher data throughput than ZigBee, and can be also easily integrated into a global network, which can be important elements of some measurement or control tasks.

## 4. Exemplary Peripheral Devices of Wireless Network

### 4.1. An Example of Implementing of The ZigBee Network in Ship Conditions

The typical use of ZigBee network is associated in most cases with the acquisition of data from sensors and measuring transducers connected to the terminal equipment of the network. The application supervising the exchange of data has been developed in the LabVIEW environment. The network consists of three types of devices: Coordinator, Router and End Device (Figure 4). In the conducted experiments, the ZigBit^TM^ modules on the base of ATmega1281V Atmel microcontroller were used, both in the form of assembled and ready-to-use ZDK MeshBean 2 boards as well as a prototype End Device. Every ZDK board can work as Coordinator, Router or End Device, what is to select using on-board DIP switches. 

Two examples of ZigBee network implementations have been elaborated in GMU. These applications, in addition to the standard acquisition of sensor data, enable control of any actuator. 

**Figure 6:**
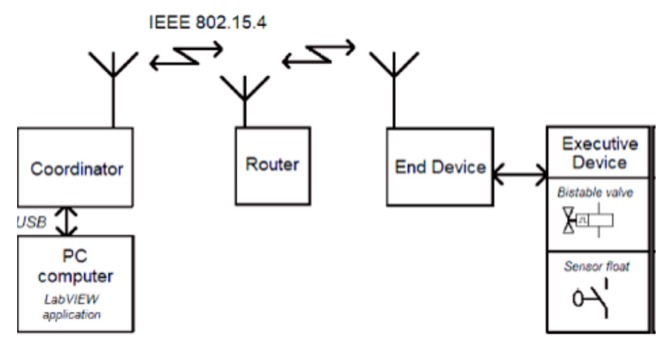
Control device in ZigBee wireless network.

In the first solution, the data from Executive Device (Figure 6), through End Device and Router, is sent wirelessly to the Coordinator that manages the entire ZigBee network, e.g. network initialization, recording of active devices and receiving data from network devices. The Coordinator communicates with the PC via a USB port. The data from the Coordinator is collected and processed in the LabVIEW application. The firmware stack on the End Device has been modified. A set of commands has been added as a support for reading the status of level sensor and identifying commands relating to the valve control module in the Executive Device (Figure 6).

**Figure 7:**
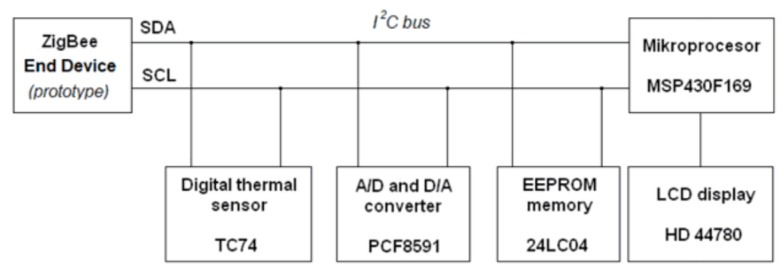
The autonomous microprocessor system cooperating with the ZigBee End Device.

The second exemplary solution, also elaborated in GMU, consists of a wireless communication channel and is supported by autonomously working microprocessor measurement and control system (Figure 7). This system communicates with the ZigBee End Device using the I^2^C bus. The prototype microprocessor system additionally contains the TC74 temperature sensor, the PCF8591 with 8-bit ADC and 8-bit DAC as well as the 24LC04 EEPROM memory connected to the I^2^C bus. The microprocessor system is connected via a 4-bit interface with an LCD display (HD 44780 controller). The elaborated peripherals can cooperate with the sensors, transducers and actuators operating as the terminals of a ship safety system. Miniature versions of the End Devices can also serve in the properly configured ZigBee mesh network as the personal identification tags.

**Figure 8:**
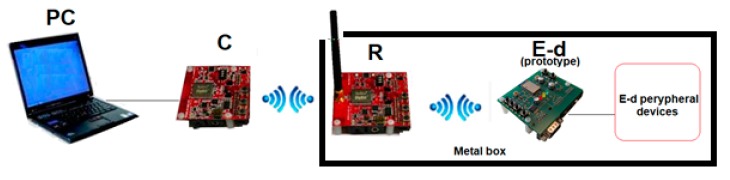
The ZigBee network testing setup.

The configuration of the tested ZigBee network is shown in Figure 8. The antenna of the ZigBee Router is placed in the gap of the metal box. 

### 4.2. Example of WiFi Network Implementation for Ship Conditions

**Figure 9:**
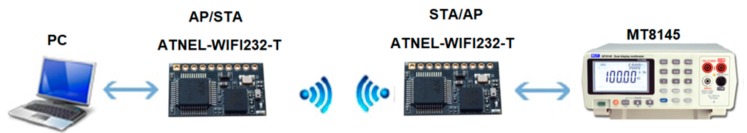
Configuration of the WiFi communication link.

The WiFi network (Figure 9), which is the subject of research, was implemented using several available systems, discussed below. The ATNEL-WIFI232-T modules [23], intended mainly for work in embedded systems, have been used to check the operation of communication of wireless devices in conditions similar to those on the ship. They enable wireless connection with devices equipped with a UART (Universal Asynchronous Receiver-Transmitter) hardware device for asynchronous serial communication over a peripheral device serial port. The UART is available on most microprocessor-based systems. RS232 or USB interfaces, controlled by UART, are the most popular as serial ports of programmable devices. All modern programmable devices, such as measuring instruments and control equipment, have one of these interfaces. Wired RS232/USB adapters are widely available. The MT8145 digital multimeter [24], as the peripheral device, was chosen for wireless communication studies. The instrument is equipped with a RS232 serial interface to connect with the ATNEL-WIFI232-T module, which communicates wirelessly with the second ATNEL-WIFI232-T module connected via an RS232/USB adapter to a PC. In Figure 9, the basic configuration of the established communication link is shown. One of the ATNEL-WIFI232-T modules was configured as an Access Point (AP) and served as a UDP (User Datagram Protocol) server, while the other was set as a workstation (STA) and its role was UDP client. Communication is fully two-sided, so the modules can be swapped with each other without having to change their configuration. 

**Figure 10:**
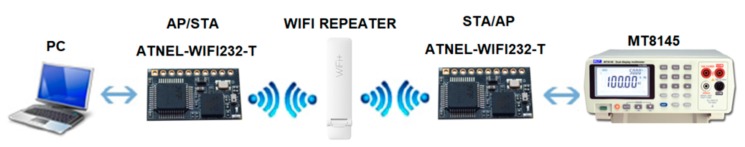
Modified WiFi communication link.

However, the considered link may not work effectively under the conditions of the ship, therefore it has been modified by enabling the WiFi repeater on the communication path (Figure 10). The conducted research used the easily available and cheap Xiaomi Mi WiFi Repeater 2 (MiR2) signal booster [25]. MiR2 has two built-in, high-performance PCB antennas to extend the router’s signal range and increase network bandwidth. In addition, it automatically selects the optimal Wi-Fi channel to provide less interference and higher download speeds of up to 300 Mb/s. MiR2 is equipped with a compact USB connector for power supply from a free USB port of PC, Power Bank or power strip with USB ports. To set up MiR2, simply download the Mi Home app, add the device and follow the intuitive on-screen instructions. The application also allows easy troubleshooting of Wi-Fi connectivity. It automatically recognizes, analyzes and solves existing network problems, as well as optimizes network channels, ensuring a permanent connection at all times. In fact, the device serves indeed as an access point, creating a new WiFi network for up to 16-users. The use of an additional device, a repeater, is intended to overcome a metal bulkhead. In this way, the WiFi connection range can be expanded with almost no limit.

## 5. Research

### 5.1. Testing The ZigBee Network Under Similar Conditions to Those on Board

As it was mentioned in Section 4, the ZigBee devices were used in the conducted experiments, both as a ready-to-use modules as well as a prototype End Device developed in GMU. They cooperated with the dedicated measurement and control microprocessor devices as the terminal devices of the network (Figure 8). 

In relation to numerous previously cited reports [9,13,14,15,16,18,19,20,21] on the results of tests on the properties of the ZigBee network on the ship and previous experience of the authors [2,4,5,6,7], the possibility of signal transmission through steel walls was tested using repeater placed in the gap made in the bulkhead.

In laboratory condition, the hermetic metal box was used to check the performance of End Device and its peripherals operations. Two experiments were carried out. In the first experiment, the End Device was placed inside the hermetic box, and the remaining part of ZigBee network was outside. In the second test, the box was equipped with a small tunnel that houses the Router (Figure 8). As expected, successful communication to End Device and its peripheral was possible only in the second experiment. In this case, the communication error rate did not differ from the value in open space. Other experiments concerned the functionality of the wireless communication channel supported by autonomously working microprocessor measurement and control system, cooperating with the ZigBee End Device. 

Considering the results shown in previous studies cited, it can be stated that the use of dedicated communication channels in steel partitions on a ship ensures the continuity of wireless communication practically on the entire ship.

### 5.2. WiFi Network Survey

**Figure 11:**
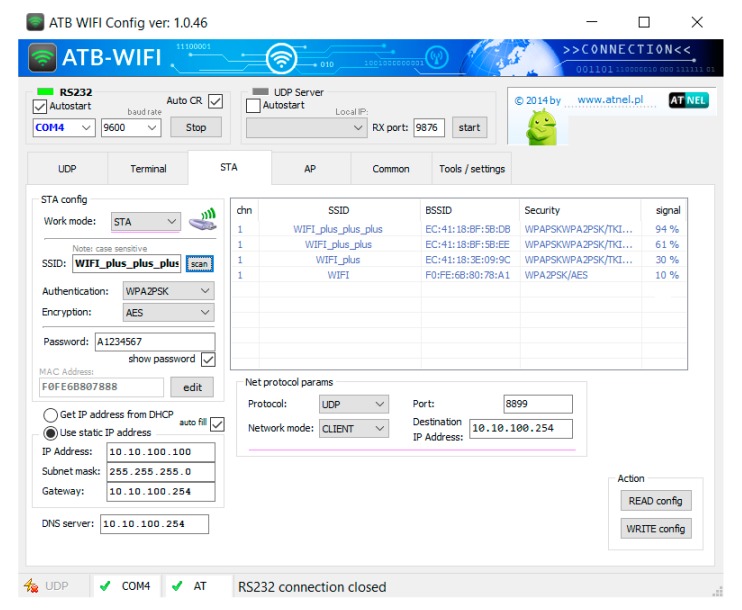
Screenshot of the ATNEL-WIFI232-T module configuration program shows WiFi network with three MiR2 routers. The source for measurement data included in Table 2 and Table 3.

**Figure 12:**
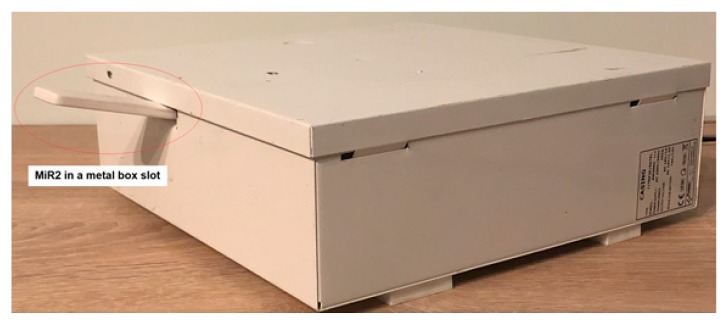
View of the metal box used in tests. The WiFi repeater is also visible in the gap made in the box.

In the research part of WiFi link, short distance tests have been carried out to check the coverage and quality of communication between two ATNEL-WIFI232-T modules (Figure 9). For all these investigations, the measurements were made for the set of 5 distances of the STA module connected to the PC from the AP module connected to the MT8145 meter, the same for each test. The values of receiver signal strength of the considered module were obtained from the WiFi module configuration application, i.e. ATB WIFI Config (Figure 11). In the first study (Figure 9), the both modules (AP and STA) operated in the open space, there were five measurements carried out for different distances The measurement results are presented in Table 2 (Test 1 column). In the second test of the communication link with the same configuration as in previous test, the AP module (together with the MT8145 meter) was placed in a closed metal housing (Figure 12). Table 2 (Test 2) presents the results of the AP strength signal measurements.

In the next 3 studies, the measurements were also carried out for previously selected five different distances of the STA module from the AP. The AP/STA transmitted signals were additionally amplified by the use of the Mi WiFi Repeater 2 (MiR2). Figure 10 presents a block diagram illustrating the tested link. Table 3 presents the results of the obtained measurement results. As previously, the signal strength values were obtained from the WiFi module configuration application, i.e. ATB WIFI Config (Figure 11), both for the AP module and MiR2 repeater. In Test 3, the AP module together with MiR2 operated in open space (Figure 11). Table 3 (Test 3 columns) contains the results of signal strength measurements made by ATB WIFI Config application, both for AP and MiR2 devices for different distances from STA module. During Test 4, the AP and MiR2 were placed inside a closed metal casing (Figure 12). The results of signal strength measurements are shown in Table 3 (Test 4 columns). The fifth test was carried out with the AP inside the metal box and MiR2 located in a gap made in the box body (Figure 12). Table 3 (Test 5 columns) shows the obtained results.

The items in Table 2 and Table 3, in which the signal strength is greater than zero, mean that it is possible to exchange data on the communication link under current conditions. The signal transmission through the metal wall is practically impossible, while the repeater placed in a small gap in the wall allows two connecting devices to communicate as in an open space, with better parameters of the transmitted signal, thanks to the properties of the repeater. 

**Figure 13:**
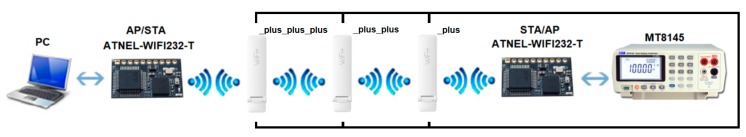
Extended wireless communication channel with three repeaters.

To ensure the effectiveness of proposed wireless channel configuration, the additional experiments were carried out (Figure 13). With the antennas of three repeaters, operating in a chain configuration, located in the gaps of three metal boxes, the obtained quality of communication was similar to the communication in the open space. The studied communication channel creates a chain structure connection, but each of the routers can additionally connect to another 15 nodes and thus a wireless tree topology can be created.

Regarding the network in this configuration, it can be stated that the use of dedicated communication channels in steel partitions, just like for the ZigBee network, ensures continuity of wireless WiFi communication practically throughout the entire ship.

### 5.3. Networks Integration

Integration of data acquisition from two different networks in a PC was carried out based on a programming environment supporting the design of control and measurement systems, NI LabVIEW. 

The application created in this environment enables control of data exchange between the controller (e.g. based on PC) and each of the tested networks. The extensive function and control libraries allow the designer to create the required data processing algorithms and to use the obtained results in the desired way, including supervision of the system from a remote location.

## 6. Concluding Remarks

This paper proposes wireless network communication-based modified implementation of ZigBee and WiFi networks with routers/repeaters playing as a wireless connectors across the steel bulkheads present on the ship and the network end-devices as a gateway between a machine (instrument, device or sensor) and ship wireless network.

In view of numerous studies ZigBee network in marine conditions, the paper presents only the results of comparative tests, which showed the effectiveness of network transmission through steel bulkheads after placing the ZigBee Router antenna in the gap in the wall, on the border of the rooms. The gap may be sealed using non-shielding materials, such as rubber or epoxy.

Comparative experiments carried out for both WiFi modules (AP and STA) operating in an open space and closed metal housing, respectively, confirm that such a wireless network implementation has limited usability in ship conditions. Moreover, in tests carried out for AP/STA modules, the transmitted signals, additionally amplified by the use of the MiR2 repeater, in the open space and using a closed metal housing, similar results were obtained as in the case of connection without a repeater. The use of the MiR2 repeater, placed properly in the socket made in the body of the box, shows that through such a communication tunnel a wireless connection with a part separated by a metal wall works similarly to connections in an open space.

In fact, all the WiFi devices as well some of the ZigBee wireless network devices, such as Coordinator or Router, need permanent cable power supply while End Devices can be battery powered. In practice, local power supply is easily available in all rooms on the ship. However, building new measurement or control channels, or modifying existing ones, is much easier in ship conditions by using of wireless networks. This technology allows flexible configuration of communication channels, easier modification and management. Also, the application of the principle of redundancy in the configuration of ship systems can be implemented in a much more efficient way using wireless networks. 

The research performed in laboratory conditions, simulating the ship obstacles well, shows usability of wireless networks for various ship applications. Although the initial tests were conducted in laboratory conditions, under these conditions the marine environment was fair faithfully recreated in terms of the presence of steel bulkheads between compartments on the ship, which was crucial for the concept of the tests carried out. This utility has been confirmed by the test results. Taking their results in connection with the results of other tests in ship conditions, it can be stated that both network standards, ZigBe and WiFi, in the appropriate configuration of network devices and software, can be used in measuring, control and signaling systems on ship. Conducted research confirmed the possibility of wireless transmission through a string of steel bulkheads, while implementing the idea presented in the paper. As a result, it can help to increase the level of safety of crew and operations on the ship.

Future works concern the integration of wireless networks, including ZibBee modules and compact communication modules working in the WiFi standard, and validation of the possibilities of their use on one of the GMU training ships. 

## Figures and Tables

**Table 1:** A list of main topics, which appeared in last years in papers dedicates to issue of maritime on board communication.

Study	Area	Author(s)
Wireless sensor networks, fundamentals and properties	Metrology and industrial applications	[4,7,10]
Study on WSN technologies for ships	Ship technology	[8,9]
Field test of wireless sensor network inside the engine room of a vessel	Ship technology	[14]
Employment of wireless sensor network for full scale ship application	Ship technology	[13,19]
ZigBee – based sensor network for shipboard environments	Ship technology	[15]
Wired sensor network on board vessels	Ship technology	[16]
A hybrid network for maritime on-board communications	Ship technology	[11,12,18]
Analysis of wired short cuts in wireless sensor networks	Ship technology	[20,21]
IEEE 802.15.4 Std	Communication technology	[17]
IEEE 802.11.Std	Communication technology	[22]

**Table 2:** The results of the AP strength signals during the tests 1 and 2.

	Test 1 *Open Space*	Test 2 *Metal Box*
Distance [m]	Signal Strength [%]	
1	49	25
2	47	3
5	21	0
7	18	0
9	3	0

**Table 3:** The results of the AP and MiR2 strength signals during the tests 3-5.

	Test 3 *Open Space* AP MiR2		Test 4 *Metal Box* AP MiR2		Test 5 *MiR2 in Gap* AP MiR2	
Distance [m]	Signal Strength [%]					
1	45	98	18	70	20	100
2	42	90	0	57	1	90
5	21	90	0	45	0	86
7	16	70	0	35	0	70
9	1	70	0	28	0	68

## Abbreviations

The following abbreviations are used in this manuscript:

ADC	Analog to Digital Converter
AP	Access Point
CRC	Cyclic Redundancy Check (Code)
DAC	Digital to Analog Converter
EEPROM	Electrically Erasable Programmable Read-Only Memory
GMU	Gdynia Maritime University
I^2^C	Inter-Integrated Circuit
IEEE	Institute of Electrical and Electronics Engineers
ISM	Industrial, Scientific and Medical
LAN	Local Area Network
LCD	Light Emitting Diode
MAC	Media Access Control
MAN	Metropolitan Area Network
MiR2	Mi WiFi Repeater 2
NI LabVIEW	National Instruments, Laboratory Virtual Instrument Engineering Workbench
PC	Personal Computer
PHY	Physical layer
STA	workstation
UART	Universal Asynchronous Receiver-Transmitter
UDP	User Datagram Protocol
USB	Universal Serial Bus
WSN	Wireless Sensor Network
